# Molecular epidemiology of enteroviruses in Cyprus 2008-2017

**DOI:** 10.1371/journal.pone.0220938

**Published:** 2019-08-08

**Authors:** Jan Richter, Christina Tryfonos, Christina Christodoulou

**Affiliations:** Department of Molecular Virology, Cyprus Institute of Neurology and Genetics, Nicosia, Cyprus; Institut Pasteur, FRANCE

## Abstract

Enteroviruses (EVs) are associated with a broad spectrum of disease manifestations, including aseptic meningitis, encephalitis, hand, foot and mouth disease, acute flaccid paralysis and acute flaccid myelitis with outbreaks being reported frequently world-wide. The aim of this study was the molecular characterization of all enteroviruses detected in Cyprus in the ten-year period from January 2008 and December 2017 as well as a description of the circulation patterns associated with the most frequently encountered genotypes. For this purpose, serum, cerebrospinal fluid, nasal swab, skin swab and/or stool samples from 2666 patients with a suspected EV infection were analysed between January 2008 and December 2017. Enteroviruses were detected in 295 (11.1%) patients, which were then investigated further for epidemiological analysis by VP1 genotyping. Overall, 24 different enterovirus types belonging to three different species were identified. The predominant species was EV-B (209/295, 71%), followed by species EV-A (77/295, 26.1%). Only one virus belonged to species EV-D, whereas EV-C enteroviruses were not identified at all. The most frequent genotypes identified were echovirus 30 (26.1%), echovirus 6 (14.2%) and coxsackievirus A6 (10.9%). While Echovirus 30 and echovirus 6 frequency was significantly higher in patients older than 3 years of age, the opposite was observed for CV-A16 and EV-A71, which dominated in young children less than 3 years. Importantly, for the current study period a significant increase of previously only sporadically observed EV-A types, such as EV-A71 and CV-A16 was noted. A phylogenetic analysis of EV-A71 showed that the majority of the EV-A71 strains from Cyprus belonged to sub-genogroup C1 and C2, with the exception of one C4 strain that was observed in 2011. The data presented provide a comprehensive picture of enteroviruses circulating in Cyprus over the last decade and will be helpful to clinicians and researchers involved in the treatment, prevention and control of enteroviral infections by helping interpret trends in enteroviral diseases by associating them with circulating serotypes, for studying the association of enteroviruses with clinical manifestations and develop strategies for designing future EV vaccines.

## Introduction

The genus Enterovirus belongs to the Picornaviridae family and is divided into 15 species, 7 of which contain human pathogenic viruses, namely the species *Enterovirus Α-D* and *Rhinovirus A-C* [1]. Enteroviruses (EVs) can be distinguished in respiratory, include all rhinovirus types, and enteric EVs, on the basis of their primary replication site. Enteric EVs are transmitted fecal-orally and replicate primarily in the gastrointestinal tract from where they can sporadically disseminate and cause infection in a wide range of other organs, including the central nervous system. Enteroviruses have long been recognized as the most common cause of aseptic meningitis, but have also been associated with a wide range of other illnesses including myocarditis, newborn sepsis, conjunctivitis, hepatitis and severe flaccid paralysis [2]. The virions possess an icosahedral symmetry and contain a single stranded positive sense RNA genome of approx. 7.5 kb length [3]. They are extremely resistant against adverse environmental conditions and disinfectants, tolerating high salinity of water as well as temperature fluctuations.

In the US, there are between 10 and 15 million new infections, giving rise to tens of thousands of hospitalizations with a seasonal peak of infection in summer and fall in temperate climates [2,4].

Outbreaks of disease caused by single genotype strains are frequently reported and represent a major public health problem [5–8]. In Southeast Asia, enterovirus A71 and coxsackieviruses A6 and A16 have been responsible for large outbreaks of hand-foot-and-mouth disease (HFMD during the last 10 to 20 years and having caused severe complications in thousands of cases with China alone exceeding more than 1 million HFMD cases annually [9]. EV-A71 has also been reported to be responsible for clusters of severe neurological illnesses in Europe [10,11] and the United States [12]. In 2014 enterovirus D68 attracted world-wide attention when it was implicated in outbreaks of severe respiratory illness and cases of acute flaccid myelitis in the United States and subsequently also other regions of the world [13–15]. A European study group comprising 17 countries subsequently showed that EV-D68 did circulate in Europe during summer and fall of 2014 [16]. The disease burden and pathogenic profile, however, was milder compared to the North-American epidemic even though the isolated viruses were genetically very similar to those the Nort-American isolates.

Previously, we had reported on the epidemiology of enteroviruses in Cyprus over the period 2003–2007 [17]. Twenty-two different serotypes had been identified in total with the main EV types echovirus 18 and echovirus 30 followed by coxsackievirus B5 and echovirus 9. Rapid changes in serotype frequency and diversity over time were observed while the overall serotype distribution corresponded with observations reported from other European countries during the same period. Aim of the current study was the molecular characterization of all enteroviruses obtained in Cyprus in the ten-year period from January 2008 and December 2017 as well as a description of the circulation patterns associated with the most frequently encountered genotypes.

## Materials and methods

### Patients and clinical samples

According to a decision taken by the Cyprus National Bioethics committee (EEBK/21.1.02.01.05), all samples taken for diagnostic purposes and have been anonymized are accessible for research without requiring neither specific written consent nor additional approval from a bioethics committee. Between January 2008 and December 2017, serum, CSF, skin swab, nasal swab and/or stool samples from 2666 patients with suspected EV infection from all districts of Cyprus were sent to our laboratory for analysis. The median patient age was 3 years ranging from one month to 77 years with a male/female ratio of 1.66. For routine laboratory diagnosis an in-house Real-Time RT-PCR assay targeting the highly conserved 5’-noncoding region was used [17]. Enterovirus positive swab, CSF and/or stool samples were then analysed further for epidemiological surveillance.

### Detection and typing

Viral RNA was extracted from 200–400μl clinical specimen (Serum, CSF, Stool, Swab) using the iPrep PureLink Virus Kit on an iPrep Purification Instrument (Thermo Fisher Scientific) according to the manufacturer’s instructions. The details of the Real-Time RT-PCR assay for routine diagnosis of enteroviruses as well as the assay for VP1 typing have been described previously [17]. In short, partial VP1 sequences of approx. 360bp length encompassing the entire hypervariable BC-loop were amplified making an unambiguously identification of the serotype possible.

Amplicons were purified using the Montage DNA Gel Extraction Kit (Millipore) and sequenced on an ABI 3500 (Applied Biosystems) in the forward and reverse directions using the respective PCR primers and the ABI BigDye V3.1 cycle sequencing kit (Applied Biosystems). Sample quality values (SQV) were assigned using the KB Basecaller (Applied Biosystems) and only sequences with SQV>25 were accepted, otherwise sequencing was repeated. Forward and reverse sequences were assembled using ClustalW and queried to sequences in GenBank using BLAST as well as the Enterovirus Genotyping Tool Version 1.0 [18,19].

### Phylogenetic analysis

All phylogenetic analyses were conducted in MEGA7 [20]. The alignment of VP1 sequences was performed using MUSCLE. Bayesian information criterion (BIC) scores were calculated for different models to determine the best fitting nucleotide substitution model. In addition, jModelTest [21] was used for evaluating the best fitting nucleotide substitution model under BIC yielding the same result (Kimura 2-Parameter model + gamma distributed rates). This model then was used to construct Maximum Likelihood (ML) phylogenetic trees with 1000 bootstrap replicates. All positions containing alignment gaps and missing data were eliminated only in pairwise sequence comparisons.

### Nucleotide sequence accession numbers

The sequences reported in this study were submitted to the GenBank sequence database under accession numbers MK111117 to MK111405.

## Results

**Fig 1:**
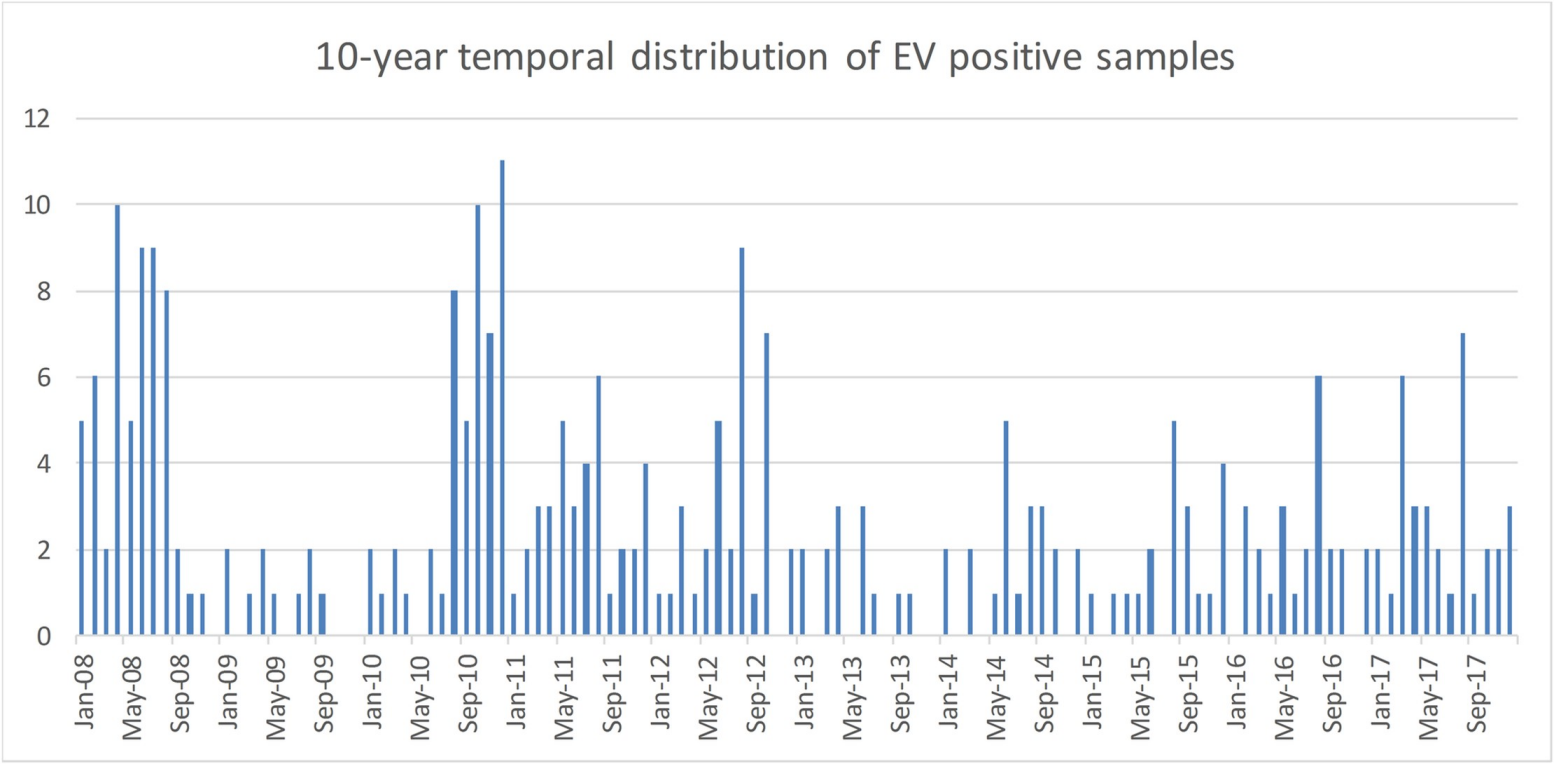
10-year monthly distribution of EV positive samples in Cyprus. Increased Enterovirus activity was usually observed during summer and early autumn.

For the 2666 patients a total of 4212 samples (2022 CSF samples, 1137 stool samples, 590 serum samples and 463 nasal/skin swab samples) were analysed. 295 patients (11.1%) were found to be enterovirus positive in at least one sample. EV genotype was determined for 287/295 samples, while 8 samples remained undetermined due to low sample concentration. The proportion of EV-positive samples per year ranged from 3.5% in 2009 up to 18.9% in 2008 with an average of 11.1% over the 10-year study period. EVs circulated throughout the year with an increased proportion detected during the summer and in early autumn. Fig 1 shows in detail the monthly distribution of enterovirus detection over the 10-year study period.

The results of the Enterovirus typing based on partial VP1 sequencing are shown in Table 1. Overall, 24 different enterovirus types belonging to three different species were identified. The predominant species was EV-B with 209 viruses distributed among 14 genotypes. 77 viruses belonged to 9 different genotypes within species EV-A. Only one virus belonged to species EV-D, whereas no EV-C enteroviruses were identified at all.

**Table 1:** Enterovirus genotypes identified between 2008 and 2017 in Cyprus. The most frequent genotype each year is indicated in bold. For 8 out of the 295 samples genotyping could not be performed due to very low sample concentration. In the last row the number of patients analysed each year is indicated.

EV species	Type	2008	2009	2010	2011	2012	2013	2014	2015	2016	2017	Total
**EV-A**	**CV-A2**	0	0	1	0	0	0	0	0	0	0	**1**
	**CV-A4**	0	0	0	0	1	1	3	0	1	0	**6**
	**CV-A5**	0	0	0	0	0	1	0	1	0	0	**2**
	**CV-A6**	0	0	8	1	**11**	0	3	3	3	3	**32**
	**CV-A8**	0	0	1	2	2	0	0	0	0	0	**5**
	**CV-A10**	0	1	1	3	0	0	0	1	0	0	**6**
	**CV-A16**	0	0	0	4	2	0	2	0	1	0	**9**
	**EV-A71**	1	2	0	1	2	0	0	2	5	1	**14**
**EV-B**	**CV-A9**	1	0	1	0	0	0	0	0	0	0	**2**
	**CV-B1**	0	0	0	0	7	0	0	0	0	0	**7**
	**CV-B2**	1	0	2	4	0	0	0	0	0	0	**7**
	**CV-B3**	0	0	0	0	0	0	0	1	0	1	**2**
	**CV-B4**	0	2	1	2	0	2	0	0	4	0	**11**
	**CV-B5**	0	**4**	1	0	2	0	2	0	**8**	0	**17**
	**E5**	0	0	0	0	1	0	0	0	0	1	**2**
	**E6**	0	0	12	4	0	0	0	**7**	2	**17**	**42**
	**E7**	0	0	0	**12**	0	0	0	0	0	0	**12**
	**E9**	0	1	3	0	0	0	2	1	0	1	**8**
	**E11**	0	0	1	1	4	0	0	0	0	1	**7**
	**E18**	0	0	0	0	0	4	0	0	0	7	**11**
	**E21**	0	0	0	0	0	0	2	1	0	0	**3**
	**E25**	2	0	0	1	0	0	0	0	0	0	**3**
	**E30**	**53**	0	**13**	0	0	**5**	**5**	0	0	1	**77**
**EV-D**	**EV-D68**	0	0	1	0	0	0	0	0	0	0	**1**
	**Untyped**	0	0	2	0	2	0	2	2	0	0	**8**
	**Total**	**58**	**10**	**48**	**35**	**34**	**13**	**21**	**19**	**24**	**33**	**295**
	**Patients analysed**	308	283	473	327	337	172	165	191	201	209	2666

The five most frequently observed enteroviruses were E30 (26.1%), E6 (14.2%), CV-A6 (10.8%), CV-B5 (5.7%) and EV-A71 (4.7%), which made up more than 60% of all enteroviruses detected. It should be noted that no polioviruses, either wild-type or vaccine-related, have been encountered reflecting the adaption of the IPV only immunization plan by paediatricians in 2006 and confirming the poliovirus-free status of Cyprus.

Regarding the diversity of the enterovirus spectrum, on average approx. 8 different genotypes were isolated in a given year (Range 5–14).

The data were then further analysed with regard to the age of the patients (Table 2).

**Table 2:** Frequency of enterovirus genotypes in persons less than 3 years old vs. persons older than 3 years. Percentages refer to the total number in each age group as shown in the header.

EV type	< = 3 years old N = 150		>3 years old N = 145	
**CV-A2**	**1**	0.7%	**0**	0.0%
**CV-A4**	**6**	4.1%	**0**	0.0%
**CV-A5**	**1**	0.7%	**1**	0.7%
**CV-A6**	**27**	18.4%	**5**	3.6%
**CV-A8**	**5**	3.4%	**0**	0.0%
**CV-A10**	**6**	4.1%	**0**	0.0%
**CV-A16**	**6**	4.1%	**3**	2.1%
**EV-A71**	**11**	7.5%	**3**	2.1%
**CV-A9**	**0**	0.0%	**2**	1.4%
**CV-B1**	**7**	4.8%	**0**	0.0%
**CV-B2**	**3**	2.0%	**4**	2.9%
**CV-B3**	**2**	1.4%	**0**	0.0%
**CV-B4**	**7**	4.8%	**4**	2.9%
**CV-B5**	**9**	6.1%	**8**	5.7%
**E5**	**1**	0.7%	**1**	0.7%
**E6**	**7**	4.8%	**35**	25.0%
**E7**	**5**	3.4%	**7**	5.0%
**E9**	**5**	3.4%	**3**	2.1%
**E11**	**6**	4.1%	**1**	0.7%
**E18**	**6**	4.1%	**5**	3.6%
**E21**	**2**	1.4%	**1**	0.7%
**E25**	**3**	2.0%	**0**	0.0%
**E30**	**17**	11.6%	**60**	42.9%
**EV-D68**	**0**	0.0%	**1**	0.7%

**Fig 2:**
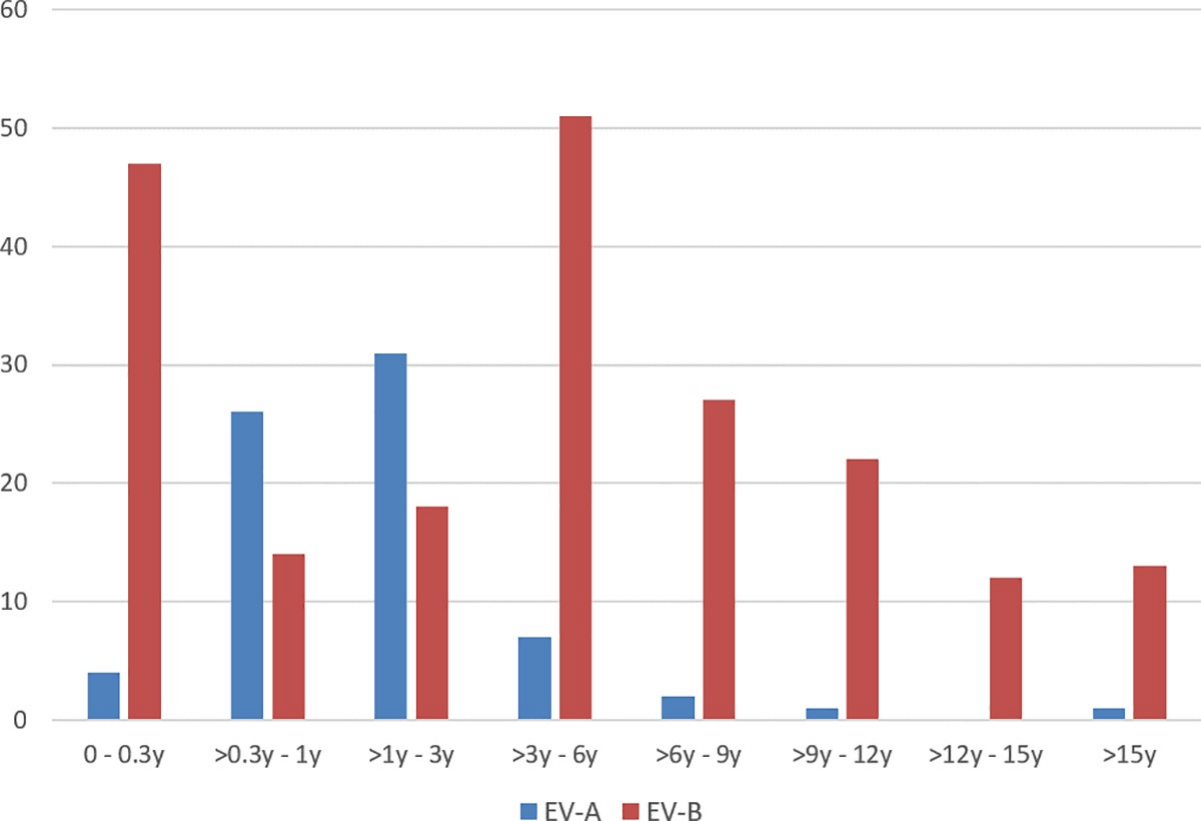
Age distribution of enterovirus species identified in Cyprus between 2008 and 2017.

In patients ≥ 3 years old (145/295) E30 and E6 were by far the most prevalent genotypes accounting for almost 70% of all positive samples, while CV-A6 and EV-A71 were the dominant genotypes in children less than 3 years of age (see Fig 2). The genotype and EV species dependent age relation is also reflected in the mean age of patients as shown in Table 3 and illustrated in Fig 2. While EV-A was found mainly in patients older than 3 months and younger than 3 years old, EV-B displayed a bi-modal distribution with one peak among neonates younger than 3 months and a second peak in children between 3 and 6 years old.

**Table 3:** Mean age of patients for the 5 most frequently observed EVs as well as the EV species.

	Mean age	95% CI
**E30**	8.53	6.4–10.7
**E6**	8.12	6.4–9.8
**CV-A6**	2.95	1.0–4.9
**CV-B5**	4.92	0.1–9.7
**EV-A71**	2.01	0.9–3.1
**EV-A**	2.50	1.6–3.4
**EV-B**	6.56	5.4–7.7

Overall, EV-A species constituted a rather small proportion in the older patient cohort (~10%), in contrast to the children ≤ 3, where they accounted for 41% of infections. The analysis also showed an increased diversity of EV genotypes in the younger children (22 genotypes) compared to those present in the older patient group (17 genotypes) over the ten-year study period (Table 2).

In order to further characterise the isolates from Cyprus and examine them in a global context, a phylogenetic analysis was performed for the five most frequently encountered genotypes.

**Fig 3:**
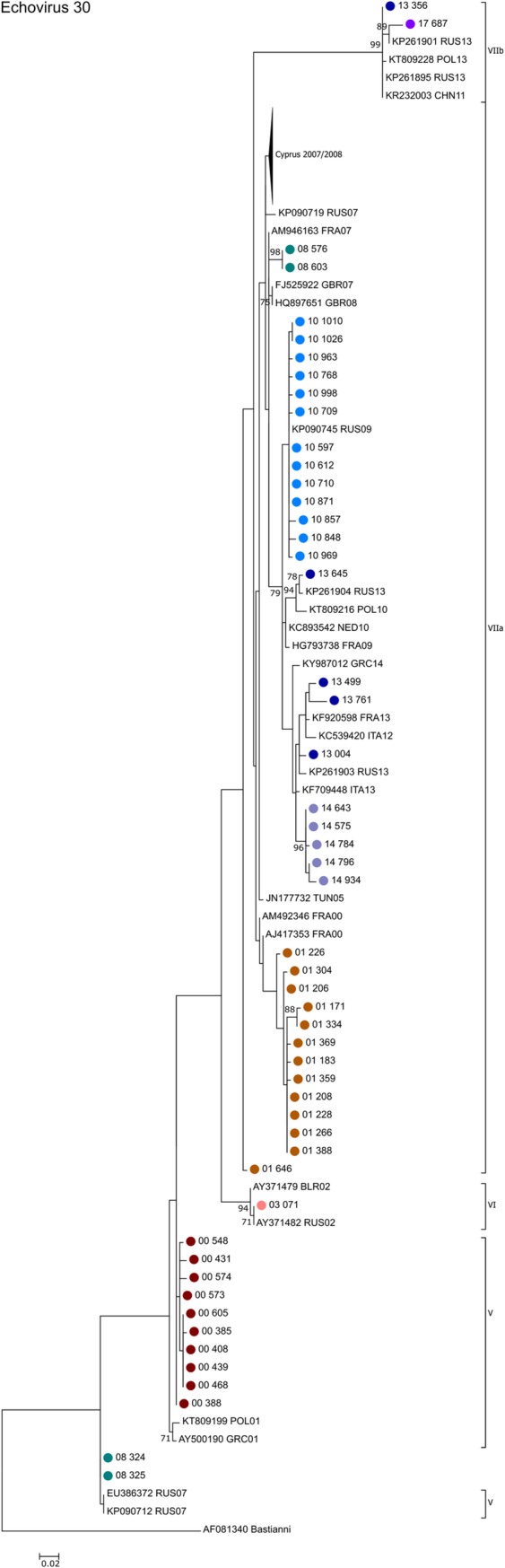
Phylogenetic analysis of partial VP1 sequences of echovirus 30 strains. Phylogenetic tree based on the partial VP1 sequence of the 77 E30 positive samples obtained in this study, E30 sequences obtained previously in Cyprus between 2000 and 2007, as well as closely related E30 sequences available in GenBank that were included in the analysis for comparison. The nodes of the Cypriot samples are color coded by year; names consist of year the taken followed by an internal laboratory code. Sequences obtained from GenBank are labelled with their accession number followed by the 3-letter country code (ISO 3166–1 alpha-3 code) and the year of isolation. The percentages of replicate trees in which the associated taxa clustered together in the standard bootstrap test (1000 replicates) are shown next to the branches. Only bootstrap values >70% are shown. Sub-genogroups assigned by the Enterovirus Genotyping Tool [19] are indicated.

Fig 3 shows the phylogenetic relationship of E30 sequences obtained between 2008 and 2017. E30 genogroups were assigned according to Savolainen-Kopra et al. [22]. The majority of the echovirus 30 strains (73/77) belonged to the most recent sub-genogroup VIIa, two samples to VIIb and two samples from 2008 to sub-genogroup V clustering closely with strains isolated from other European countries, especially from Russia, during the same years, which is consistent with previous observations of large-scale emergence and turnover of E30 variants with detection of related viruses from widely separated countries [23]. It is also evident that in 2008 and 2013 viruses belonging to different sub-genogroups co-circulated in Cyprus.

**Fig 4:**
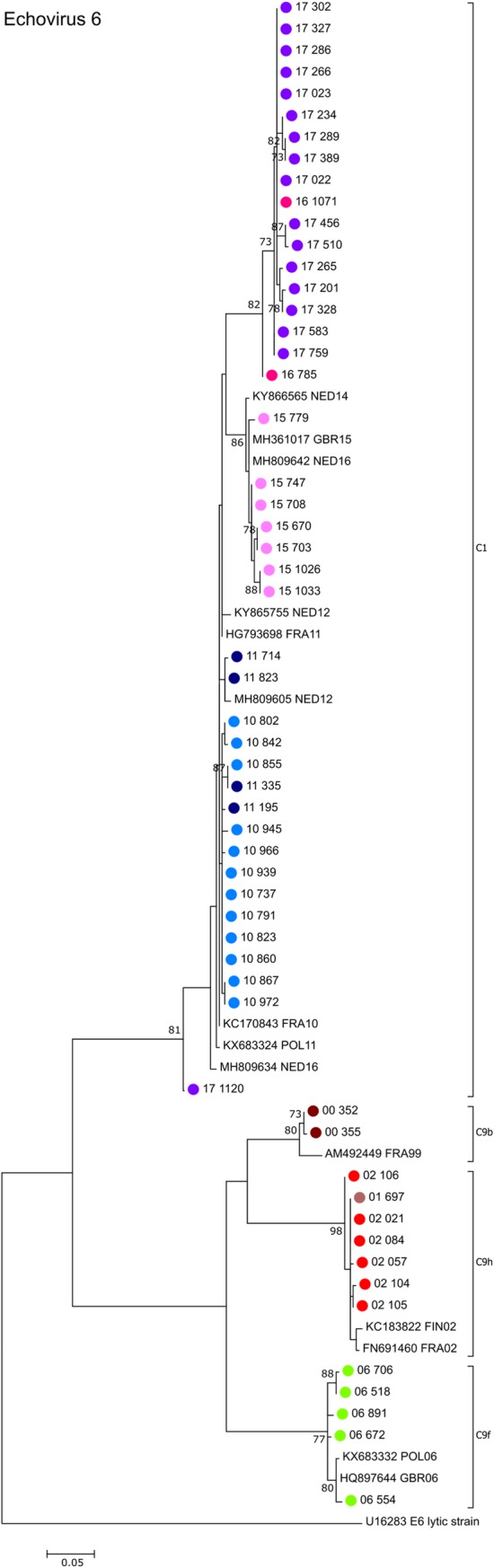
Phylogenetic analysis of partial VP1 sequences of echovirus 6 strains. Phylogenetic tree based on the partial VP1 sequence of the 42 E6 positive samples obtained in this study, E6 sequences obtained previously in Cyprus between 2000 and 2007, as well as closely related E6 sequences available in GenBank that were included in the analysis for comparison. Sequences obtained from GenBank are labelled with their accession number followed by the 3-letter country code (ISO 3166–1 alpha-3 code) and the year of isolation. The percentages of replicate trees in which the associated taxa clustered together in the standard bootstrap test (1000 replicates) are shown next to the branches. Only bootstrap values >70% are shown. Sub-genogroups were assigned according to the classification applied by Cabrerizo et al [25].

All echovirus 6 sequences from Cyprus obtained in this study period belong to sub-genogroup C1 displaying a ladder-like evolution, while those obtained in previous studies all belonged to sub-genogroup C9 clustering into three distinct sublineages (9b, 9f, 9h) according to their year of isolation (Fig 4). While the 2010 and 2015 samples cluster very closely with isolates obtained in the Netherlands, Poland, France or Great Britain of the same years, the 2016/2017 samples did not have closely related matches in GenBank differing by at least 6% on the nucleotide level.

**Fig 5:**
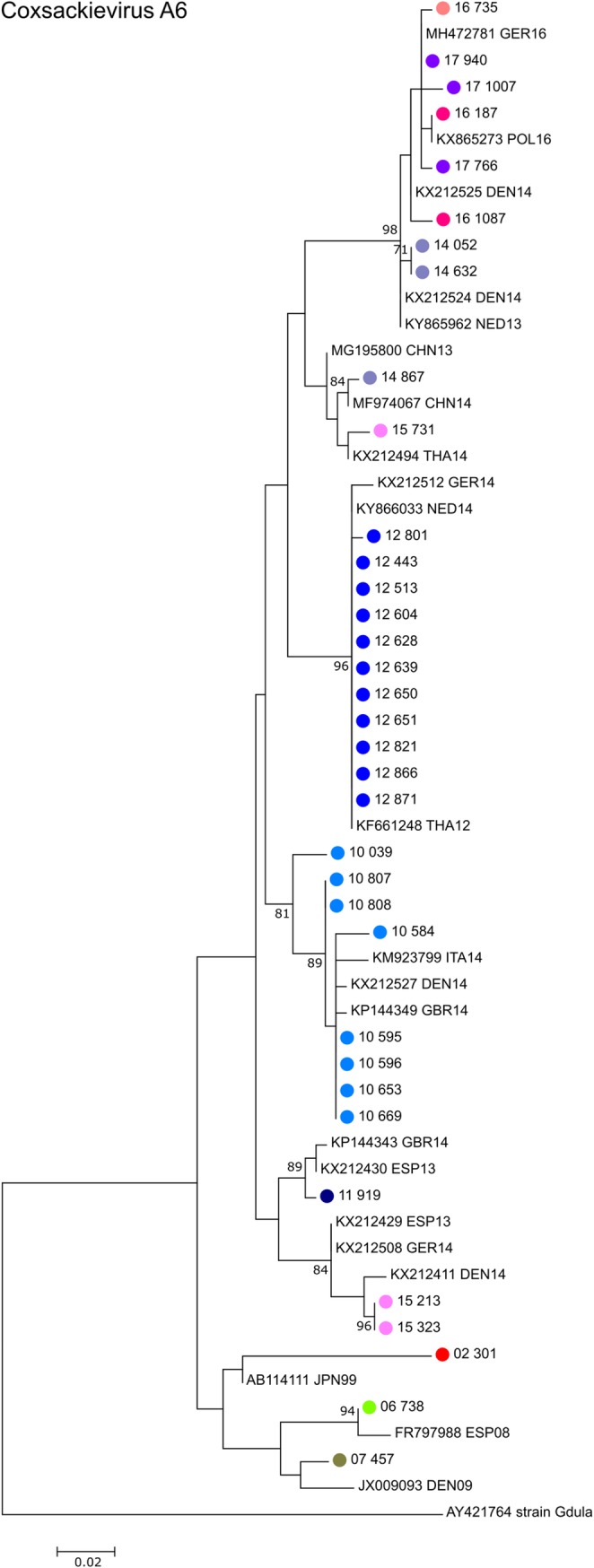
Phylogenetic analysis of partial VP1 sequences of coxsackievirus A6 strains. Phylogenetic tree based on the partial VP1 sequence of the 32 CV-A6 positive samples obtained in this study, CV-A6 sequences obtained previously in Cyprus between 2000 and 2007, as well as closely related CV-A6 sequences available in GenBank that were included in the analysis for comparison. Sequences obtained from GenBank are labelled with their accession number followed by the 3-letter country code (ISO 3166–1 alpha-3 code) and the year of isolation. The percentages of replicate trees in which the associated taxa clustered together in the standard bootstrap test (1000 replicates) are shown next to the branches. Only bootstrap values >70% are shown.

Regarding coxsackievirus A6, sequences clustered mainly monophyletic according to their year of isolation (Fig 5). There was a close temporal relationship to CV-A6 isolates recovered in other countries in Europe with the exception of two samples co-circulating in 2014 and 2015, which were closely related to strains observed in China and Thailand during that period.

**Fig 6:**
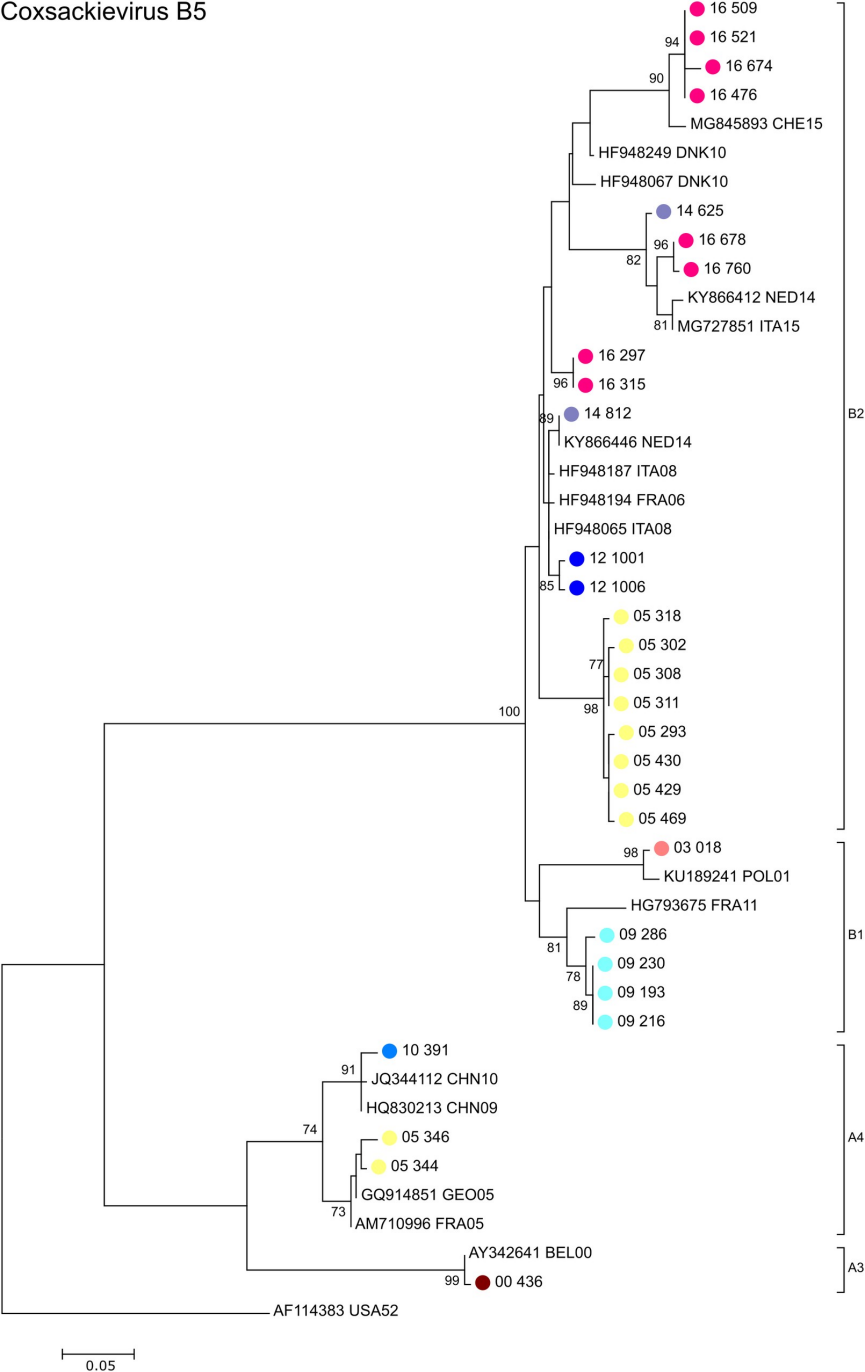
Phylogenetic analysis of partial VP1 sequences of coxsackievirus B5 strains. Phylogenetic tree based on the partial VP1 sequence of the 17 CV-B5 positive samples obtained in this study, CV-B5 sequences obtained previously in Cyprus between 2000 and 2007, as well as closely related CV-B5 sequences available in GenBank that were included in the analysis for comparison. Sequences obtained from GenBank are labelled with their accession number followed by the 3-letter country code (ISO 3166–1 alpha-3 code) and the year of isolation. The percentages of replicate trees in which the associated taxa clustered together in the standard bootstrap test (1000 replicates) are shown next to the branches. Only bootstrap values >70% are shown. Sub-genogroups were assigned according to the classification introduced by Henquell et al [24].

Fig 6 shows that the majority of CV-B5 sequences obtained in Cyprus belong to sub-genogroup B2 according to the classification introduced by Henquell et al [24] and closely matched with other European isolates. Only one sample from 2010 was identified as sub-genogroup A4 that was highly similar to Chinese isolates from the same year.

**Fig 7:**
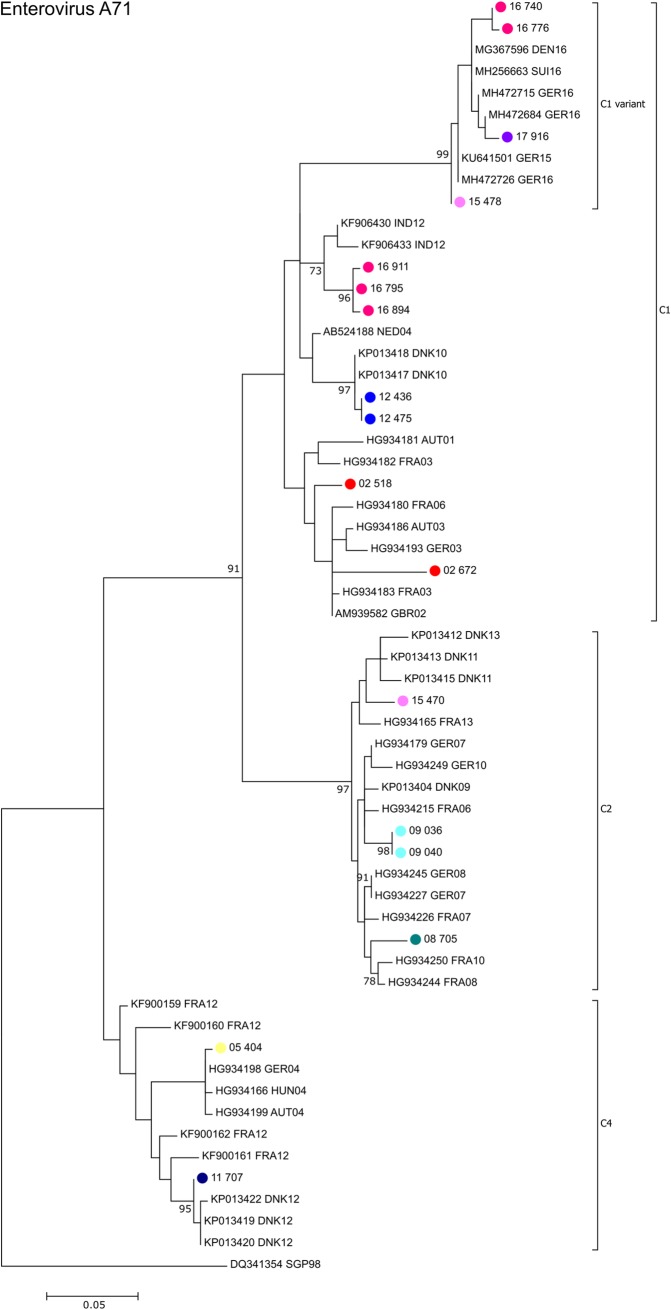
Phylogenetic analysis of partial VP1 sequences of enterovirus A71 strains. Phylogenetic tree based on the partial VP1 sequence of 14 EV-A71 positive samples detected in Cyprus between 2008 and 2017, three EV-A71 samples detected between 2000 and 2007 that had been reported previously as well as closely related EV-A71 sequences available in GenBank that were included in the analysis for comparison. Sequences obtained from GenBank are labelled with their accession number followed by the 3-letter country code (ISO 3166–1 alpha-3 code) and the year of isolation. The nodes of the Cypriot samples are color coded by year; names consists of year the taken followed by an internal laboratory code. The percentages of replicate trees in which the associated taxa clustered together in the standard bootstrap test (1000 replicates) are shown next to the branches. Only bootstrap values >70% are shown. Sub-genogroups assigned by the Enterovirus Genotyping Tool [19] are indicated.

With regard to EV-A71, the majority of the strains belonged to sub-genogroup C1 and C2, with the exception of one C4 strain that was observed in 2011 (Fig 7). Most of the EV-A71 strains from Cyprus clustered closely with other European isolates of the same time period with the exception of two C1 samples of 2016, which had the closest matchings in GenBank for strains from India from 2012. The 2015, 2016 and 2017 EV-A71 sub-genogroup C1 strains from Cyprus cluster closely with a new recombinant strain identified in Germany in 2015 that had been labelled C1 variant and was found in Spain 2016 during an EV-A71 outbreak that was associated with severe neurological disease.

## Discussion

In the current study echovirus 30 was the most frequently detected enterovirus accounting for 20% (57/295) of all EV positive samples. In 4 out of the 5 years, when it was detected, it was the predominant type (2008, 2010, 2013 and 2014).

Echovirus 30 regularly causes large outbreaks of meningitis worldwide and was shown to circulate with a pattern of successive dominant lineages that reach a global prevalence with a 3- to 5-year turnover and replacement in Europe [26–28].

A similar pattern was also observed previously in Cyprus, where it caused outbreaks in 2000/2001 and 2007 with almost no detection in between [29,30]. Similar outbreaks of E30 have been observed in Europe during the same decade, such as in Finland in 2009 [6] in Greece and Italy in 2012 [31,32], in Germany in 2008 and 2013 [33], the UK in 2008 and 2013 [34] and notably in 5 European countries in 2018 [35]. It is worth noting that with regard to the age of the patients, in our study E30 was mainly identified in patients older than 3 years old (60 out of 77, 78%). A similar E30 dominance in older patients had previously been reported also from the UK and Switzerland [34,36].

Echovirus 6 is one of the most frequently identified EV types in Europe and worldwide and usually associated with outbreaks or sporadic cases of aseptic meningitis [34,37–41]. In the current study, E6 was the second most prevalent enterovirus overall and the most prevalent type in the years 2015 and 2017. A similar increase in E6 incidence was seen in the Netherlands in 2016 [42]. As noticed for E30, a significant higher frequency of echovirus 6 was observed in the patient group older than 3 years (35 out of 42, 83%).

Long known to be one of the main pathogens responsible for causing HFMD outbreaks, in Asia enterovirus CV-A6 has in recent years replaced EV-A71 and CV-A16 as the primary pathogen causing HFMD [43–45]. Before 2008, A6 was considered a rare enterovirus in European countries including Cyprus, however, since then an increasing activity is evident with outbreaks reported from Finland [46] France [47] and Spain [48]. In Cyprus in the 10-year period investigated, a strong increase in the number of CV-A6 positive cases was observed being detected in 7 out of the 10 years and being the predominant type in 2012 compared to our previous studies covering the period 2000–2007, where the virus was detected only sporadically [17,29]. Even though in other countries coxsackievirus A6 was characterized by a high incidence in adults [46], the majority of CV-A6 in our study were found in children less than 3 years of age (27 out of 32, 84%).

Coxsackievirus B5 is frequently reported in virus isolation and regularly appears in the top five commonly identified EV types in the United States, China and Europe being commonly associated with acute viral meningitis [37,49–51]. It was the fourth most frequent genotype detected and the predominant enterovirus in 2009 and 2016. In the years between, however, it was only sporadically detected. Previously, an analysis of the phylodynamic pattern of coxsackieviruses B5 circulation showed the existence of two CV-B5 genogroups with opposed evolutionary pathways as a result of immune selection pressure [26]. An important feature of CV-B5 transmission pattern was the co-circulation of virus strains of multiple subpopulations resulting in a fluctuation of the predominant genogroup in the epidemic waves. This was also evident in a previous study, where we had analyzed enteroviruses in sewage samples in Cyprus and had observed that different strains of CV-B5 were continuously circulating in the population without being detected in clinical samples during the same time period [30]. With regard to age, there was no significant difference in the detection frequency in younger vs. older patients.

EV-A71 and CV-A16 are the most important causative agents of hand, foot, and mouth disease, however, EV-A71 can also cause a diverse range of neurological diseases, including brainstem encephalitis and neurogenic pulmonary edema and is now considered as the primary etiological agent for acute flaccid paralysis [52]. Compared to the previous studies conducted in Cyprus [17,29] there was a significant increase in EV-A71, which was detected in 7 out of the 10 years investigated. The majority of the EV-A71 strains from Cyprus were identified belong to sub-genogroup C1 and C2, with the exception of one C4 strain in 2011. In Spain an outbreak of EV-A71 that was associated with severe neurological disease was observed in 2016 [53]. Most of the isolates had been classified as sub-genogroup C1 that was phylogenetically related to a new recombinant variant strain associated with severe neurological diseases in Germany and France in 2015 and 2016. EV-A71 strains from Cyprus In the Asia-Pacific region and especially China, large outbreaks of HFMD caused by EV-A71 are observed regularly, especially by sub-genogroup C4 strains [9,54,55]. In Europe an increase in the otherwise rarely seen EV-A71 sub-genogroup C4 strain was reported in France and Denmark in 2012 [56,57] and Russia in 2013 [58], but has not been continued afterwards so far.

## Conclusions

In conclusion, this study provides a comprehensive picture of enteroviruses circulating in Cyprus over the last decade. A genotype distribution that corresponded closely with observations in other European countries during the same period was observed, which is plausible given the large number of tourists (>2.5 million) that visit the island each year compared to the number of inhabitants (~840,000).

A significant increase in EV-A incidence, namely coxsackievirus A6 and enterovirus A71, was noted. As research and development of HFMD vaccines has so far primarily focused on EV-A71 and CV-A16 vaccines, the emergence of CV-A6 as a major causative agent for HFMD may require a rapid shift towards appropriate intervention strategies.

The Global Disease Detection Operations Center at the CDC in the US included EV-A71 in the top-five global infectious disease threats, due to its potential to cause large outbreaks and severe, life threatening neurologic disease [59]. Continued monitoring and surveillance are therefore crucial to elucidate and recognize enteroviral infections and the changes in EV circulation and epidemic cycles.
